# Experimental knee pain impairs submaximal force steadiness in isometric, eccentric, and concentric muscle actions

**DOI:** 10.1186/s13075-015-0768-1

**Published:** 2015-09-12

**Authors:** David A. Rice, Peter J. McNair, Gwyn N. Lewis, Jamie Mannion

**Affiliations:** Health and Rehabilitation Research Institute, Auckland University of Technology, Private Bag 92006, Auckland, 1142 New Zealand; Waitemata Pain Service, Department of Anaesthesia and Perioperative Medicine, North Shore Hospital, Private Bag 93-503, Takapuna, Auckland, New Zealand; Unitec Institute of Technology, Private Bag 92025, Victoria St West, Auckland, New Zealand

## Abstract

**Introduction:**

Populations with knee joint damage, including arthritis, have noted impairments in the regulation of submaximal muscle force. It is difficult to determine the exact cause of such impairments given the joint pathology and associated neuromuscular adaptations. Experimental pain models that have been used to isolate the effects of pain on muscle force regulation have shown impaired force steadiness during acute pain. However, few studies have examined force regulation during dynamic contractions, and these findings have been inconsistent. The goal of the current study was to examine the effect of experimental knee joint pain on submaximal quadriceps force regulation during isometric and dynamic contractions.

**Methods:**

The study involved fifteen healthy participants. Participants were seated in an isokinetic dynamometer. Knee extensor force matching tasks were completed in isometric, eccentric, and concentric muscle contraction conditions. The target force was set to 10 % of maximum for each contraction type. Hypertonic saline was then injected into the infrapatella fat pad to generate acute joint pain. The force matching tasks were repeated during pain and once more 5 min after pain had subsided.

**Results:**

Hypertonic saline resulted in knee pain with an average peak pain rating of 5.5 ± 2.1 (0–10 scale) that lasted for 18 ± 4 mins. Force steadiness significantly reduced during pain across all three muscle contraction conditions. There was a trend to increased force matching error during pain but this was not significant.

**Conclusion:**

Experimental knee pain leads to impaired quadriceps force steadiness during isometric, eccentric, and concentric contractions, providing further evidence that joint pain directly affects motor performance. Given the established relationship between submaximal muscle force steadiness and function, such an effect may be detrimental to the performance of tasks in daily life. In order to restore motor performance in people with painful arthritic conditions of the knee, it may be important to first manage their pain more effectively.

## Introduction

Globally, the prevalence of knee osteoarthritis (OA) ranges from approximately 6 to 25 % in those aged 60 years or older and has a marked impact on function and quality of life [1–3]. Similar to other chronic pain conditions, people with knee OA experience deficits in strength and motor control in the muscles surrounding the painful joint [4–7]. While impairments in quadriceps strength are well documented in knee OA [5, 8], deficits in submaximal muscle force production and regulation have received less attention. The regulation of submaximal muscle force is more relevant for functional tasks that are encountered in everyday activities, such as ascending or descending stairs, squatting, or stepping over objects.

There is some evidence of impaired submaximal muscle force regulation in individuals with knee joint OA [9, 10]. Compared with healthy control subjects, Hortobargyi et al. [10] noted force appreciation deficits in individuals with OA who commonly experienced pain categorised as slight to moderate in the week prior to testing and during the testing session. Some indirect evidence for the influence of pain is highlighted in Smith et al. [9], who observed an improvement in force steadiness following knee joint replacement. The clinical importance of these findings is highlighted by relationships that have been demonstrated between submaximal muscle force steadiness and functional performance, including walking endurance [11], stair-climbing ability [12], and a history of falling [13].

In chronic conditions such as arthritis, identifying the cause of impaired muscle force regulation can be difficult given the probable presence of joint afferent damage, pain, swelling, and muscular changes such as disuse atrophy. To isolate the impact of pain itself, acute experimental pain models have been used to examine how force control is modified without the confounding effect of other pathology-related factors. These studies have consistently shown a reversible increase in force variability and force-matching error during pain [14–17]. Interestingly, despite the importance of particularly eccentric but also concentric muscle action to efficient and smooth movement, only two studies have focused on such modes of muscle action. One of these studies targeted the upper-limb muscles, which may have different physiological pathways operating for control of muscle action compared with postural muscles such as the quadriceps in the lower limb. That study examined both isometric and dynamic (eccentric and concentric) muscle contractions, and found that acute shoulder pain impaired force variability during isometric contractions only, and not during dynamic contractions [17]. This finding is in contrast to individuals with knee OA, where greater impairments in submaximal quadriceps force regulation were observed during dynamic muscle contractions [10]. Given this information, it would be pertinent to more closely examine the impact of knee pain on submaximal quadriceps force production during both static and dynamic muscle contractions.

The goal of the current study was to examine the effect of experimental knee pain on submaximal force-matching ability at the knee. Knee extensor force-matching tasks were undertaken during isometric, eccentric, and concentric muscle contractions. The study hypothesised that force-matching ability and force steadiness would diminish during acute knee pain and that this would be evident during both static and dynamic contractions.

## Methods

### Participants

Fifteen healthy individuals (mean age 28 years, range 18–49 years; eight females) were recruited for the study. Based on the work of Hortobargyi et al. [10] and our pilot work, sample size calculations indicated that, with a power of 0.8 and an alpha level set to 0.05, 15 subjects were required to observe a 20 % difference across no pain and pain conditions. Participants were required to have no history of knee joint pathology or injury, an absence of pain in the lower limb, and no neuromuscular impairments of either lower limb. The study was granted ethical approval from the UNITEC Institute of Technology and the Auckland University of Technology ethics committees, and all participants provided informed consent prior to involvement in the study.

### Maximum voluntary contraction

Participants were seated in a Biodex dynamometer (Biodex Medical Systems Inc, Shirley, New York, USA) with the right leg securely fastened into a lever arm pad with the axis of rotation aligned with the centre of the knee joint. The hips were positioned at 90° and the thigh was securely strapped to the chair. To establish the isometric maximum voluntary contraction (MVC), the knee joint was positioned in 60° of flexion. For establishing eccentric and concentric MVC, the knee was rotated through 0° (full extension) to 100° of flexion, or vice versa, at 10°/second. Participants were instructed to contract the quadriceps muscle as hard as possible for 6 seconds (isometric) or for the duration of the movement (eccentric, concentric). Three repetitions were completed for the isometric contraction and five repetitions for eccentric and concentric contractions. MVC was established as the peak torque during any of the trials.

### Force-matching task

Participants were seated as already described. For each type of contraction, the target force to match was set at 10 % of MVC. This value was chosen because it is utilised in tasks that require fine/controlled movements of the lower limb (e.g. driving a car, stepping on stairs, or operating machinery with foot pedals). Participants were provided with the target force together with their actual knee extensor force on a monitor located directly in front of them. The computer monitor was positioned 1.5 metres from the subject at shoulder height, and slightly to the left to allow movement of the dynamometer lever arm. The scale of the *y* axis of the graph was set to fill the computer screen, with the uppermost visible point being 50 % of the MVC for each subject. Participants were asked to match the target force as accurately as possible, and, once the target force level was achieved, to keep their force as steady as possible at that level for the duration of the task.

In the isometric force-matching condition, each trial lasted approximately 10 seconds. In the eccentric force-matching condition, the knee was rotated from 0 to 100° of flexion at 10°/second (10 seconds trial duration). In the concentric force-matching condition, the knee was rotated from 100 to 0° at 10°/second (10 seconds trial duration). Pilot work identified that at an angular velocity of 10°/second participants have sufficient time to make adjustments to their force levels during the range of motion from 0 to 100°. At greater angular velocities (e.g. 30°/second), because participants have just over 3 seconds to complete the task, this often invoked ‘reactive responses’ leading to notable overshooting, particularly in the early part of the range of motion, and little time for correction. Force data were collected at 2000 Hz using a PowerLab (ML785; ADInstruments Pty Ltd., Bella Vista, NSW, Australia) data acquisition unit and LabChart 7 software (ADInstruments Pty Ltd., Bella Vista, NSW, Australia).

### Experimental pain

Experimental knee pain was induced by injecting 5.8 % hypertonic saline into the infrapatellar fat pad. With the knee resting in slight flexion, 1 ml hypertonic saline was injected into the fat pad of the right knee with a 27 gauge needle mounted on a 1 ml syringe. Injections were from a medial approach with the needle inserted ~1 cm at a 45° angle in a posterolateral direction. All injections were performed without local anaesthesia, under sterile conditions. This type of injection routinely generates knee pain for up to 20 minutes.

### Protocol

Participants attended a familiarisation session no more than 7 days prior to the test session. At the familiarisation session, participants had the procedure explained to them and completed three quadriceps MVCs in each contraction condition (isometric, eccentric, concentric). Participants then completed 15 practice force-matching trials at 10 % MVC for each of the three contraction types. The reliability of the tests has been established by Hortobargyi et al. [10] and previous pilot work in our laboratory that generated intraclass correlation coefficients greater than 0.80.

**Fig. 1:**
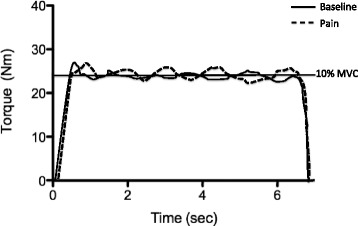
Typical example of data for a ‘no pain’ and ‘pain’ condition during isometric muscle activation. *MVC* maximum voluntary contraction

At the test session, participants first completed three MVC contractions for the isometric condition and five for the concentric and eccentric conditions. A 1 minute rest period was given between each MVC. Ten minutes after the completion of MVCs, five force-matching trials were completed in each of the three contraction conditions (baseline), with a 1–2 second gap between trials. The order of the three contraction conditions was randomised among participants. Acute knee pain was then induced following injection of hypertonic saline. Approximately 30 seconds after needle withdrawal, participants repeated the force-matching trials in the same order as previously (pain). Numerical pain ratings were obtained every 90 seconds using a 0 (no pain) to 10 (worst pain imaginable) scale. Five minutes after the knee pain had subsided to 0 on the pain scale, participants completed the force-matching trials once more in the same order as previously (post pain). Figure 1 shows a schematic of data recorded for an isometric muscle action at baseline prior to the injection and during pain.

### Data processing and analysis

Data were processed using Signal software (Cambridge Electronic Design, Cambridge, UK). For all contraction conditions, the first trial was discarded and the central 6 seconds of data (20–80° of motion) were analysed for the remaining four trials. Two variables were of interest. The standard deviation across the 6 seconds of data provided a measure of steadiness across the response signal of the participant without reference to the target force level. In contrast, the root mean square (RMS) takes into account the target force level, and is calculated by subtracting the force signal generated by the subject from the target/reference force. These two outcome measures were averaged across the four trials completed in each contraction condition.

Group data were analysed using a two-way repeated-measures analysis of variance with the factors of contraction (isometric, concentric, eccentric) and pain (baseline, pain, post pain). Significant main and interaction effects were investigated using independent-sample or paired *t* tests, as appropriate. For these *t* tests, the percent error rate was calculated, which provides the proportion of significant results that could occur as a result of chance. The level of significance was set at 0.05.

## Results

Data were collected from 14 participants. One participant had a vasovagal response after the hypertonic saline injection and was not able to complete the remaining tests.

The average peak pain rating following the injection was 5.5 ± 2.1 and lasted 18 ± 4 minutes after needle withdrawal. Analysis of MVC data indicated significant differences among the three contraction types (*F*_2,26_ = 33.7; *P* <0.001). The MVC during eccentric contractions was significantly higher than during isometric and concentric contractions (both *P* <0.001), and the MVC during isometric contractions was greater than that during concentric contractions (*P* = 0.006).

### Force-matching error

**Fig. 2:**
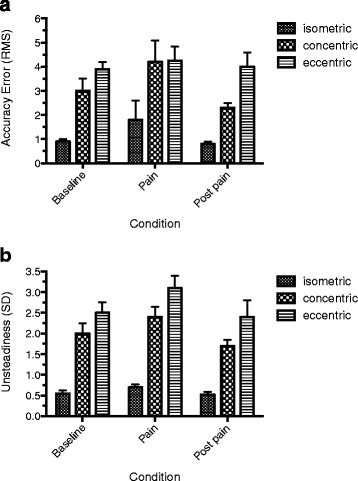
Group data showing **a** matching/accuracy error and **b** steadiness across pain conditions during the different muscle activation modes. *RMS* root mean square, *SD* standard deviation

Figure 2a shows group RMS error data at baseline, during pain, and post pain for the three contraction conditions. There was a main effect of contraction (*F*_2,26_ = 25.7; *P* <0.001), with further testing indicating that the RMS error was significantly lower during isometric contractions compared with both eccentric and concentric contractions (both *P* <0.001). There was no difference in the RMS error between eccentric and concentric contractions (*P* = 0.08). The main effect of pain (*F*_2,26_ = 3.3; *P* = 0.09) and the pain by contraction interaction effect (*F*_4,52_ = 1.1; *P* = 0.4) were not significant.

### Force steadiness

Figure 2b shows group force standard deviation data at baseline, during pain, and post pain for the three contraction conditions. Both the main effects of contraction (*F*_2,26_ = 43.0; *P* <0.001) and pain (*F*_2,26_ = 12.3; *P* <0.001) were significant. Force standard deviation was significantly higher during the eccentric and concentric contractions compared with isometric contractions (both *P* <0.001), and was higher during eccentric contractions compared with concentric contractions (*P* = 0.01). In addition, force standard deviation was significantly higher during pain compared with baseline (*P* = 0.02) and with post pain (*P* < 0.001). Force standard deviation also was significantly higher at baseline compared with post pain (*P* = 0.03). The interaction effect between contraction and pain was not significant (*F*_4,52_ = 2.9; *P* = 0.06).

Overall, across both force-matching and force steadiness analyses, the percentage error rate associated with multiple *t* tests was 6 %.

## Discussion

The main finding of this study is a reversible impairment in submaximal quadriceps force steadiness during experimental knee pain that was consistent across isometric, eccentric, and concentric contractions. While there was a trend towards increased quadriceps force-matching error during pain, this did not reach significance. Thus, during pain, the participants were still able to match the target force but were significantly less steady in maintaining the target level of muscle force. This study therefore supports previous studies that have shown impairments in isometric force steadiness during acute pain, but shows for the first time that this also occurs in eccentric and concentric muscle contractions during experimental knee pain.

### Force steadiness

Increased force fluctuations are commonly described both in acute experimental pain [15–17] and in chronic pain conditions [9, 10, 18]. Salomoni and colleagues [15, 16] have shown that this increase in variability occurs in multiple axes and not just the primary axis of force generation. The purported mechanisms for this increase in variability include alterations in motor unit recruitment and firing rates, increased activation of synergist and antagonist muscles, impaired proprioceptive information, and alterations in spinal interneuron modulation of motoneuron firing.

It has been theorised that reduced force steadiness is related to fluctuations in muscle recruitment and greater co-contraction of antagonist muscles [19, 20]. In support of this theory, reduced torque steadiness in children with cerebral palsy has been related to variability in muscle activation and the level of antagonist co-activation [21]. However, none of the studies using acute or chronic pain models to investigate force steadiness reported any differences in either agonist or antagonist muscle activation during pain. One study investigating knee extensor force steadiness post anterior cruciate ligament (ACL) reconstruction [22] found that increased hamstring activation was associated with reduced force-matching error, providing further evidence against this theory. Reports of increased tangential force displacement [15, 16, 23] and increased motor unit synchronisation [14] during acute pain raise the possibility of an altered, less efficient motor unit recruitment strategy in response to pain. However, Muceli et al. [18] demonstrated no difference in the variability of motor unit firing rates in patients with chronic neck pain despite greater force fluctuations. Instead, these authors speculated that the greater force variability was more probably due to altered proprioceptive input. Proprioceptive and somatosensory processing is known to be impaired in many chronic pain conditions [24–27]. It is also known that nociceptive information converges on spinal interneurons which also receive input from other joint and muscle receptors [28]. Increased nociceptive input thus has the potential to modify sensory information synapsing on these interneurons, impairing sensorimotor integration and increasing force fluctuations.

Irrespective of the potential mechanisms, our findings have important clinical implications because quadriceps force steadiness is related to functional performance in individuals with knee OA [10] and hip OA [12], and impaired quadriceps force steadiness has been shown to differentiate older adults with a history of falling from those who do not fall, despite similar levels of activity and muscle strength [13]. While previous studies in older adults have shown that quadriceps force steadiness can be improved by both strength training [29, 30] and task-specific training [31], our findings suggest that joint pain may directly impair force steadiness in individuals with knee injury or pathology. To restore motor performance in these patients, it may be important to first manage their knee pain more effectively.

### Force-matching error

The RMS error data showed a trend towards greater error during pain, although this did not reach significance. This observation implies that the participants were still able to accurately generate the target level of force despite increased variability. Fewer studies have reported data related to absolute error during force-matching tasks. Hortobagyi et al. [10] reported more than twice as much error in eccentric and concentric force-matching tasks in a knee OA population compared with healthy controls, although no impairment was detected during isometric force matching. The ability to adequately match force during pain may be related to the use and dominance of visual feedback to complete the task. During the force-matching tasks, visual information on the target force is provided. In most situations, participants appear able to use this visual information for force matching during pain even when there are impairments in the proprioceptive information. It would be interesting to determine whether force could be graded as accurately in the absence of visual feedback.

### Limitations

A possible limitation of the current work is that acute pain is known to reduce muscle strength, and the increased variability during pain might possibly be due to the participants working at a relatively higher level of maximum contraction during pain. However, previous studies in chronic pain populations have found equivalent impairments in force steadiness at both absolute and relative (% MVC) force targets [18]. Also, impairments in muscle strength and force-matching ability are not related in individuals who have recently undergone knee joint arthroplasty [9] and ACL repair [22]. These findings suggest that increased force variability during acute pain is independent of strength impairments. Another possible limitation is that hypertonic saline injection produces transient knee pain that may not accurately mimic the pain experienced by clinical populations as a result of knee injury or pathology. While the pain induced by the injection is temporary, it was induced in structures known to be an important source of nociception in clinical conditions [32–35]. It should also be noted that neuroplasticity in central nervous system pathways associated with chronic pain have additional potential for disrupting sensory feedback that might further disrupt performance in force steadiness and accuracy.

## Conclusion

This study demonstrated that experimental knee pain leads to impaired quadriceps force steadiness during isometric, eccentric, and concentric contractions, providing further evidence that joint pain directly affects motor performance. Such an effect may be detrimental in the performance of tasks in daily life. Furthermore, if not addressed adequately, this effect has the potential to slow rehabilitation involving re-education of such tasks. In future, the neural mechanisms linking activation of the nociceptive system with reduced force steadiness should be clearly discerned. This may help in developing more effective rehabilitation strategies that target the key mechanisms which perpetuate chronic pain’s negative interaction with motor performance in those with arthritic conditions.

## Abbreviations

ACL: Anterior cruciate ligament
MVC: Maximum voluntary contraction
OA: Osteoarthritis
RMS: Root mean square
